# A Switch in the Mechanism of Communication between the Two DNA-Binding Sites in the SfiI Restriction Endonuclease

**DOI:** 10.1016/j.jmb.2007.08.030

**Published:** 2007-11-09

**Authors:** Stuart R.W. Bellamy, Susan E. Milsom, Yana S. Kovacheva, Richard B. Sessions, Stephen E. Halford

**Affiliations:** The DNA–Protein Interactions Unit, Department of Biochemistry, School of Medical Sciences, University of Bristol, University Walk, Bristol BS8 1TD, UK

**Keywords:** AUC, analytical ultracentrifugation, DTT, dithiothreitol, HEX, hexachlorofluorescein, KNF, Koshland–Nemethy–Filmer, LIN, linear, *M*_r_, relative molecular mass, MWC, Monod–Wyman–Changeux, OC, open circle, SC, supercoiled, wt, wild type, allostery, cooperativity, DNA–protein interaction, protein–protein interaction, restriction enzyme

## Abstract

While many Type II restriction enzymes are dimers with a single DNA-binding cleft between the subunits, SfiI is a tetramer of identical subunits. Two of its subunits (a dimeric unit) create one DNA-binding cleft, and the other two create a second cleft on the opposite side of the protein. The two clefts bind specific DNA cooperatively to give a complex of SfiI with two recognition sites. This complex is responsible for essentially all of the DNA-cleavage reactions by SfiI: virtually none is due to the complex with one site. The communication between the DNA-binding clefts was examined by disrupting one of the very few polar interactions in the otherwise hydrophobic interface between the dimeric units: a tyrosine hydroxyl was removed by mutation to phenylalanine. The mutant protein remained tetrameric in solution and could bind two DNA sites. But instead of being activated by binding two sites, like wild-type SfiI, it showed maximal activity when bound to a single site and had a lower activity when bound to two sites. This interaction across the dimer interface thus enforces in wild-type SfiI a cooperative transition between inactive and active states in both dimers, but without this interaction as in the mutant protein, a single dimer can undergo the transition to give a stable intermediate with one inactive dimer and one active dimer.

## Introduction

Type II restriction endonucleases recognise specific sequences in DNA, typically palindromic sites 4 to 8 bp long, and cut the DNA at specified positions within or close to the site.^1^ Their reactions usually (but not always^2^) require Mg^2+^ as a cofactor.^3^ Some Type II endonucleases are dimers of identical subunits that interact symmetrically with their palindromic sites.^4^^,^^5^ These have a single DNA-binding cleft at the subunit interface, and they act at individual copies of their target sites.^6–8^ The best-studied restriction enzymes, such as EcoRV and BamHI,^8–14^ and those most widely used *in vitro* as tools for molecular biology,^15^ all function in this manner. Consequently, these are often considered as “standard” restriction enzymes. However, many Type II endonucleases differ from the standard in that they are fully active only after interacting with two copies of their recognition site.^16–20^ The Type II enzymes that need two sites fall into two subtypes, IIE or IIF.^21^ The Type IIE restriction enzymes bind two (or more^22^) copies of their recognition sequence yet cleave only one.^23^ They contain two DNA-binding clefts, catalytic and allosteric, but the catalytic cleft is inactive unless cognate DNA is also bound at the allosteric site.^17^^,^^22^ In contrast, the Type IIF enzymes form complexes with two DNA sites, at equivalent loci in the protein,^24^^,^^25^ and then cut both sites in a concerted reaction.^26–30^

The first restriction enzyme found to act concertedly at two DNA sites was the SfiI endonuclease.^26^^,^^27^ SfiI recognises the sequence GGCCNNNN↓NGGCC (where N is any base and ↓ is the point of cleavage),^31^ but it cleaves substrates with two copies of this sequence more rapidly than DNA with a single copy. Moreover, in steady-state reactions at low enzyme concentrations, SfiI converts DNA with two sites directly to the final product cut at both sites, without liberating intermediates cut at one site:^26^^,^^27^ the intermediates remain bound to the enzyme until it has cut both strands at both sites.^32^ On DNA with two sites, SfiI loops out the DNA between the sites,^33–35^ but it can also bind simultaneously to two separate DNA molecules that each have one copy of the recognition sequence.^36^^,^^37^ The rates of their reactions on one-site substrates increase sigmoidally with DNA concentration, indicative of positive cooperativity.^36^ The interaction with two sites is obligatory, as virtually no DNA is cleaved by any complex of SfiI with a single recognition site.^36,38^ Many genetic events—such as DNA replication and recombination, and the regulation of gene expression—often depend on proteins interacting with two sites at separate locations in the DNA, and the SfiI restriction enzyme has become one of the principal test systems for analysing the mechanisms of long-range communications between distant DNA sites.^39^

Many restriction enzymes are now known to belong to the Type IIF family: examples include Cfr10I,^28^ NgoMIV,^24^ Bse643I,^40–42^ BspMI,^29^ SgrAI,^43^^,^^44^ Mly113I and BbeI,^19^ and almost all of the Type II enzymes that cut DNA bilaterally on either side of their recognition sites, such as BcgI and AloI.^20^ All of these enzymes need to interact with two recognition sites for full activity. Proteins that interact with two DNA sites generally prefer sites in *cis*, on the same molecule of DNA, to sites in *trans*, on separate DNA molecules, simply because two sites in *cis* will almost always be in closer proximity than sites in *trans*.^39^^,^^45^ Consequently, all of these enzymes are capable of cleaving DNA with two sites more rapidly than DNA with one site.

**Fig. 1:**
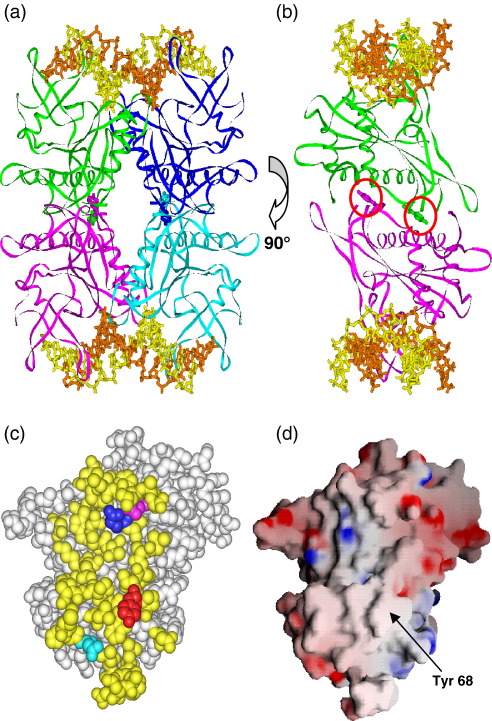
SfiI structures. (a) The crystal structure of tetrameric SfiI endonuclease bound to two specific DNA duplexes (RCSB Protein Data Bank, accession code 2EZV).^25^ Two polypeptide chains (represented as green and blue ribbons) form a primary dimer bound to one DNA, and the other two (magenta and cyan ribbons) form a second dimer bound to another DNA. In both duplexes, the two strands are in orange and yellow. (b) Side-on view. The structure of the SfiI–DNA complex in (a) has been rotated through 90°, and the two monomers in blue and cyan, which would be at the front of this view, have been removed for clarity. In both (a) and (b), the Tyr68 residues at the interdimer interface are shown in ball-and-stick format and, in (b), are highlighted with red circles. (c) Space-filling representation of the surface of the magenta-coloured monomer in (b) that faces the monomer in green across the dimer interface. All of the residues in yellow lie within 5 Å of the green monomer. Residues that make side-chain-to-side-chain interactions with the opposite monomer are coloured as follows: Tyr68, red; Gln30, purple; Gln3, light blue; Gln26, dark blue. (d) Electrostatic potential^48^ on the protein surface shown in (c). Positively charged residues are in blue, negatively charged residues are in red and uncharged residues are in white. The arrow marks Tyr68.

Most Type IIF enzymes, including SfiI, are known from analytical ultracentrifugation (AUC) studies to exist in solution as tetramers of identical subunits.^26^^,^^28^^,^^29^^,^^34^ [SgrAI is an exception, but while it is a dimer in solution, two dimers bound to separate sites associate to form a tetramer before cutting DNA.^44^] In the crystal structures of the tetrameric enzymes, two of the subunits (a primary dimer) constitute one DNA-binding cleft, and the other two subunits constitute a second identical cleft.^24^^,^^25^^,^^28^^,^^40^ The two primary dimers are arranged back-to-back, so that their DNA-binding clefts are on the opposite sides of the protein (Figure 1(a) and (b)). The primary dimers in SfiI are comparable to the dimeric restriction enzyme BglI,^46^ an enzyme whose recognition site, GCCNNNN↓NGGC, is a truncated SfiI site. The structures of the individual subunits of SfiI and the arrangement of the two subunits in its primary dimer are similar to those in BglI, likewise its motifs for DNA sequence recognition and catalysis.^47^ The subunit interface within the dimer has, however, a much smaller area in SfiI than in BglI. In addition, SfiI and BglI have very different surfaces opposite the DNA-binding cleft: polar and solvent-exposed in the case of dimeric BglI,^46^ but almost completely hydrophobic and buried in tetrameric SfiI.^25^

In previous studies, the mode of communication between the two DNA-binding clefts in another tetrameric Type IIF enzyme, Bse634I, was examined by mutating selected amino acids at the dimer–dimer interface.^41^^,^^42^ A mutation that converted the tetramer into a dimer yielded an enzyme with much the same properties as a standard dimeric restriction enzyme: while wild-type (wt) Bse634I cleaves DNA with two target sites at a rapid rate and DNA with one site at a slow rate, the dimeric mutant cleaved both substrates at equally rapid rates.^41^ Another mutant at the dimer interface of Bse634I cleaved both one-site and two-site substrates at diminished rates, while a third cleaved both substrates at elevated rates, but in both cases the two-site DNA was still cleaved more rapidly than the one-site DNA.^42^ In this study, we report on the effect of a single amino-acid substitution at the dimer–dimer interface of the SfiI endonuclease, a conservative change from a tyrosine to a phenylalanine that removes just one hydroxyl group per subunit. In contrast to the previous mutants of Bse634I, this mutation yields an enzyme that cleaves DNA with one cognate site more rapidly than DNA with two sites. It thus switches SfiI from an enzyme that is activated by binding two DNA sites to one that shows its maximal activity on binding a single site.

## Results

### Subunit communications in SfiI

In the crystal structure of SfiI bound to two copies of its recognition sequence, two subunits (a primary dimer) bind one duplex, with each monomer contacting one of the two GGCC elements of the sequence.^25^ The other two subunits bind the second duplex on the opposite side of the tetramer in a back-to-back arrangement (Figure 1(a) and (b)). The interface between the two monomers within each dimer has a surface area (1650 Å^2^) that is not only smaller than that for the related dimeric enzyme BglI (3500 Å^2^)^46^ but which is also smaller than those for other tetrameric restriction enzymes (viz. 3100 Å^2^ for Bse634I).^40^ Many of the amino acids at the interface within each dimer are polar in nature and interact with the opposite monomer.^25^

In contrast, the subunit interactions at the interface between the primary dimers have a larger contact area in SfiI than in the other tetramers (3450 Å^2^ compared to 1700 Å^2^ for Bse634I). In SfiI, the inter-dimer interface is composed almost entirely of nonpolar residues:^25^ the amino acids in one subunit that lie within 5 Å of the opposite subunit across the dimer interface (Figure 1(c)) coincide with a patch on this surface that is devoid of either positively or negatively charged side chains^48^ (Figure 1(d)). The subunit interactions across the inter-dimer interface are thus mainly van der Waals contacts between hydrophobic side chains, although they also include some contacts between peptidyl main-chain carbonyl and amino groups. However, there appear to be only two side-chain-to-side-chain hydrogen bonds across the inter-dimer interface: between Gln3 in one subunit and Gln26 in the opposite partner, likewise between Tyr68 and Gln30 (Figure 1(c)). Gln3 is located at the outside edge of the inter-dimer interface (Figure 1(c)) while Tyr68 is positioned near the centre of the contact area (Figure 1(c) and (d)). Moreover, the aromatic side chain of Tyr68 protrudes from the surface of each subunit into a pocket in the opposite subunit (Figure 1(b)). Given the paucity of directional interactions across this interface, it seemed plausible that Tyr68 might play a pivotal role in the communication between the two DNA-binding clefts. To test this possibility, the hydroxyl moiety from the Tyr68 side chain was removed by using site-directed mutagenesis to replace the tyrosine with phenylalanine to yield the mutant Y68F.

### Enzyme stability and quaternary structure

DNA-cleavage reactions of the Y68F mutant were initially carried out at 50 °C (the standard temperature for SfiI assays) in the same way as for wt SfiI: the enzyme was diluted into dilution buffer (Materials and Methods), incubated at 50 °C and then added to a solution of DNA and MgCl_2_.^26^^,^^32^ However, Y68F lost activity rapidly between dilution and subsequent addition to the DNA: after 2 and 5 min in dilution buffer at 50 °C, its activity had fallen by factors of 10 and 1000, respectively, while the wt enzyme retained full activity (data not shown). However, when the Y68F enzyme was mixed with the DNA substrate before initiating the reaction with MgCl_2_, it retained full activity. For wt SfiI, no differences were observed between reactions initiated by adding diluted enzyme to the mix of DNA and MgCl_2_ and reactions initiated by adding MgCl_2_ to premixed enzyme and DNA. All subsequent analyses of Y68F were therefore carried out in the presence of cognate DNA, and all DNA-cleavage reactions were initiated by adding Mg^2+^ to mixtures of enzyme and DNA.

Since the Y68F protein is unstable in the absence of DNA, its oligomeric state was determined with DNA present. The DNA was a 21-bp duplex with the recognition sequence for SfiI, HEX-21 (Table 1). This duplex carries a chromophoric label, hexachlorofluorescein (HEX), attached to the 5′ end of the “top” strand. HEX-21 has an absorbance peak at 539 nm, a wavelength at which neither protein nor nucleotides have any intrinsic absorbance.

**Fig. 2:**
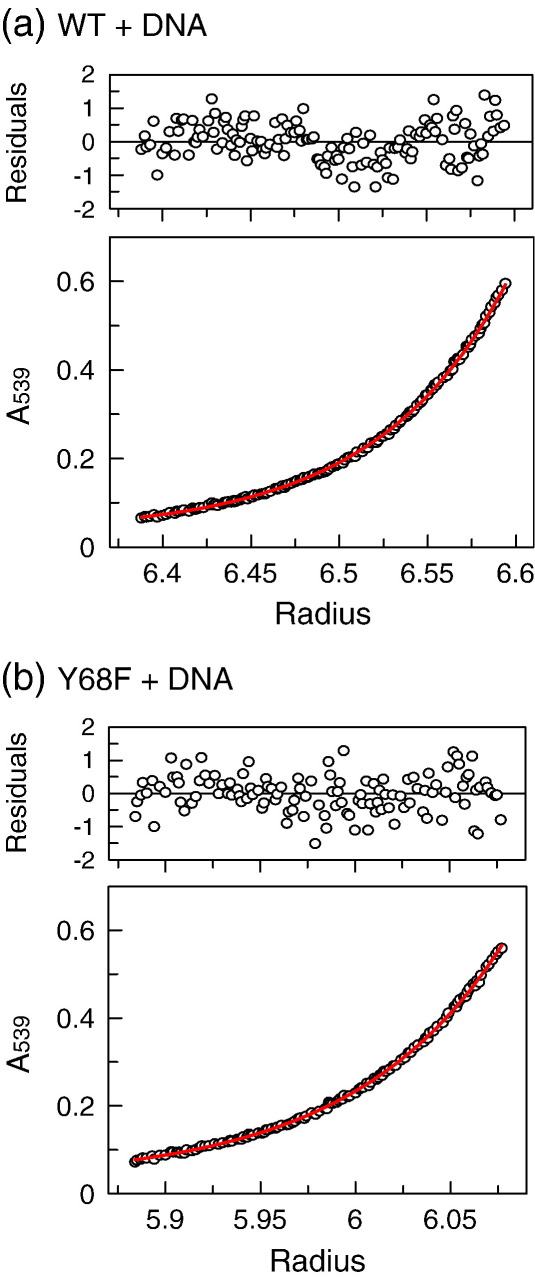
Sedimentation equilibrium. Samples contained 2.5 μM enzyme ((a) wt SfiI; (b) Y68F) and 2.5 μM DNA (HEX-21) in AUC buffer at 20 °C. The two main panels show the differences in absorbance at 539 nm between sample and reference channels as a function of centrifugal radius, after centrifugation for 16 h at 10,000 rpm at 20 °C. The red lines through the experimental points correspond to the best global fit of the data at varied speeds to the equation for a single species.^49^ The best fits to the data for the complexes of HEX-21 with wt SfiI (a) and with Y68F (b) gave *M*_r_ values of 154,203 and 156,274, respectively. The upper panels show the distribution of the residuals between data and optimal fits.

The sedimentation of HEX-21 to equilibrium was measured by recording in the AUC the absorbance of the samples at 539 nm as a function of centrifugal radius. The samples comprised HEX-21 alone or HEX-21 with either Y68F or wt SfiI (the DNA was present at half the concentration of DNA-binding sites in the enzyme). In all three cases, the increase in absorbance with centrifugal radius was fitted to the equation for a single homogenous species^49^ to yield apparent *M*_r_ (relative molecular mass) values. By itself, HEX-21 gave an *M*_r_ of about 16,000, close to that expected for a HEX-labelled DNA of 21 bp (data not shown). The best fits to the data in the presence of wt SfiI or Y68F gave *M*_r_ values of 154,203 and 156,274, respectively (Figure 2), which are both close to that predicted for the SfiI tetramer bound to two duplexes (151,367).

The removal of the hydroxyl group from the side chain of Tyr68, by replacing it with Phe, diminished the stability of the protein: upon dilution to low protein concentrations, the mutant lost activity, possibly due to subunit dissociation at low concentrations. Nevertheless, as with wt SfiI, the *M*_r_ of Y68F bound to a 21-bp DNA matched that expected for the tetramer bound to two duplexes.

### DNA-binding studies

**Fig. 3:**
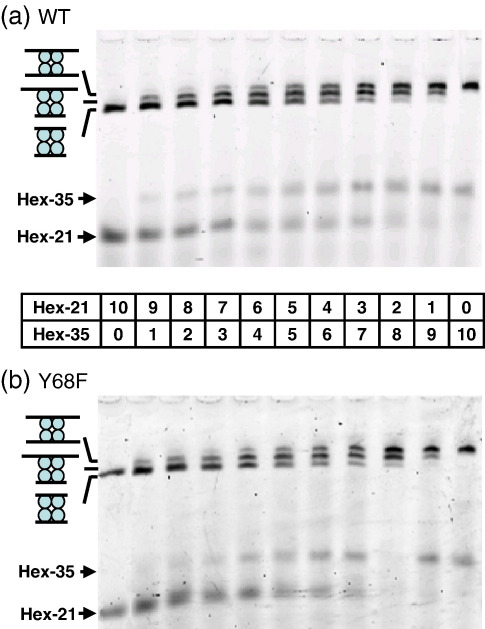
DNA binding. Samples in binding buffer contained 5 nM enzyme ((a) wt SfiI; (b) Y68F) and DNA (total concentration 10 nM). The DNA was HEX-21 alone (left-hand lane), HEX-35 alone (right-hand lane) or mixtures of HEX-21 and HEX-35 (central lanes), in ratios varying from 9:1 to 1:9 as indicated. After 30 min at room temperature, the samples were subjected to electrophoresis through polyacrylamide, and the gels were analysed in a PhosphorImager to record the HEX fluorescence. The electrophoretic mobilities of free HEX-21 and free HEX-35 are marked by arrows on the left of the gel. The mobilities of three DNA–protein complexes are also marked by the cartoons on the left: in order of decreasing mobility, the SfiI tetramer bound to two molecules of HEX-35; to one molecule of HEX-35 and one of HEX-21; to two molecules of HEX-21.

Gel retardation was used to compare the DNA–protein complexes formed by Y68F and by wt SfiI (Figure 3). As in previous studies,^36^ two duplexes of different lengths were employed: HEX-21, the 21-bp DNA used in the AUC, and an elongated version, HEX-35 (Table 1). The complexes of enzyme bound to either duplex, or to both, were separated from each other, and from the free duplexes, by electrophoresis through polyacrylamide. The DNA was detected by HEX fluorescence.

The two duplexes were added, at a constant total concentration, to fixed amounts of either Y68F or wt SfiI, to give mixtures that contained twice the molarity of duplex over enzyme tetramer. The binding buffer^36,37^ contained Ca^2+^: this ion can promote specific binding by Mg^2+^-dependent enzymes without supporting DNA cleavage.^10–12^ The addition of HEX-21 alone to either protein gave a single retarded complex, as did the addition of HEX-35 alone (Figure 3, left-hand and right-hand lanes, respectively). The complex with HEX-21 had a faster electrophoretic mobility than that with HEX-35. When both duplexes were added, three complexes were observed: one with the same mobility as that with HEX-21, one equal to the complex with HEX-35 and a third with an intermediate mobility (Figure 3, central lanes). The yields of the three complexes varied with the ratio of the two duplexes in a binomial manner, with the 1:2:1 distribution occurring when the ratio of the concentrations of HEX-21 to HEX-35 was close to 1:1. The same pattern was observed with Y68F.

The intermediate complex, with a mobility in between that with HEX-21 alone and that with HEX-35 alone, must contain one molecule of HEX-21 and one molecule of HEX-35. The complexes with the highest and the lowest mobilities therefore correspond, respectively, to the SfiI tetramer bound to two molecules of HEX-21 and to two molecules of HEX-35. Both Y68F and wt SfiI can thus form complexes that contain two DNA duplexes bound to a tetrameric protein. However, both proteins were saturated at the DNA concentrations used here, so it remains to be determined whether there are any differences in affinity and/or cooperativity between wt and mutant proteins.

### Cleavage of two-site plasmid

**Fig. 4:**
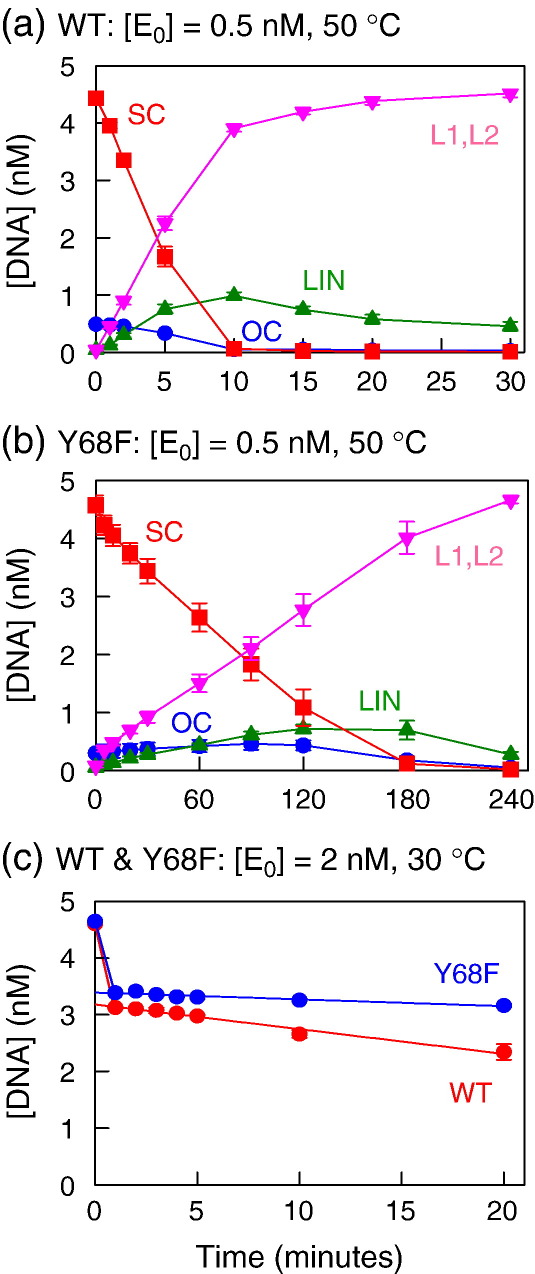
Two-site reactions. (a) and (b) The reactions contained, in reaction buffer at 50 °C, 5 nM [^3^H]pGB1 (a plasmid with two SfiI sites, initially 90% SC monomer) and 0.5 nM enzyme: (a) wt SfiI; (b) Y68F. The reactions were started by adding MgCl_2_ to the mixture of enzyme and DNA. Aliquots were taken at various times after adding Mg^2+^ and analysed by electrophoresis through agarose. The amounts of the following forms of DNA were determined: intact SC DNA substrate, red squares; nicked OC DNA, blue circles; linear DNA cut at one SfiI site (LIN), green triangles; average of the two linear products after cutting both SfiI sites (L1 and L2), purple inverted triangles. (c) The reactions contained, in reaction buffer at 30 °C, 5 nM pGB1 and 2 nM enzyme: wt SfiI, red circles; Y68F, blue circles. The reactions were carried out as described above. Only the concentration of the SC substrate is shown.

The optimal substrates for SfiI are supercoiled (SC) plasmids that have two copies of its recognition sequence.^27^ A single tetramer of SfiI binds to two sites in *cis* and traps the intervening DNA in a loop.^33–35^ It then cleaves all four of its target phosphodiester bonds before dissociating from the DNA: the initial product liberated from the enzyme is the final product cut in both strands at both sites, to the virtual exclusion of intermediates cut at one, two or three bonds.^32^ To find out if the Y68F mutant acts like wt SfiI in this respect, the reactions of both enzymes were examined on a plasmid with two SfiI sites, pGB1.^26^ The reactions were initially conducted under steady-state conditions, with [*E*_0_] < [*S*] (Figure 4(a) and (b)), to be able to observe directly the nature of the DNA liberated from the enzyme, rather than the enzyme-bound intermediates generated during the reaction.

Under these conditions, wt SfiI converted almost all of the SC plasmid directly to the two linear products (L1 and L2) cut in both strands at both sites: none of the open circle (OC) form of the DNA, cut in one strand, and only a small amount of the full-length linear (LIN) form of the DNA, cut in both strands at one site, were released from the enzyme during the reaction (Figure 4(a)). To allow comparisons between substrates with one SfiI site and substrates with two SfiI sites, all steady-state rates are recorded here in terms of moles of SfiI sites cleaved per mole of enzyme tetramer per minute (mol/mol/min). The velocity of wt SfiI on this two-site plasmid was 2.2 mol/mol/min (Figure 4(a)), the *V*_max_ for this reaction.^26^^,^^27^ The Y68F mutant also cleaved the two-site plasmid in a highly concerted manner: it liberated very little of either the nicked OC form or the LIN product cut at one site. Instead, it converted this DNA directly to the final products cut at both sites, L1 and L2 (Figure 4(b)). However, the rate at which Y68F cleaved pGB1 (0.11 mol/mol/min) was 20 times slower than wt SfiI.

It is unlikely that the 20-fold difference is due to a difference in *K*_m_ as the gel-shift experiments (Figure 3) had shown that the DNA concentrations used here were sufficient to saturate either enzyme. However, the rate-limiting step for the complete reaction pathway for wt SfiI (the process that determines its steady-state velocity) is the final dissociation of the products cut at both sites: all of the preceding stages, including the four DNA-cleavage steps, are relatively rapid.^32^ The reduction in reaction velocity could thus be due to either product release from Y68F being 20-fold slower than from wt, or the rates of the DNA-cleavage steps in its reaction pathway being reduced to values below that for product release by wt SfiI. These possibilities can be distinguished by determining whether Y68F generates a pre-steady state burst of product formation. If the DNA cleavage steps are faster than product release, the enzyme will create a burst of enzyme-bound product, to a concentration equal to that of the enzyme, prior to a slow steady-state phase, the rate of which is limited by product release.^50^ On the other hand, if the DNA-cleavage steps are rate-limiting for Y68F, there will be no burst, and the reaction will proceed instead at the linear steady-state rate from time zero.

The reactions of both wt and mutant enzymes were studied under conditions where the presence of a burst phase is readily detected: with an enzyme concentration (2 nM) approaching that of the DNA (5 nM) so that the enzyme-bound product can constitute a significant fraction of the total product, and at a reaction temperature (30 °C) where SfiI has a very slow turnover rate^32^, thus allowing the reactions at elevated enzyme concentrations to still be monitored. Under these conditions, both wt and Y68F enzymes cleaved a fraction of the two-site substrate rapidly before entering a slower phase during which the concentration of the substrate declined linearly with time: the decline was more rapid with wt SfiI than with Y68F (Figure 4(c)). Hence, on a DNA with two sites, both enzymes are rate-limited by product release, but Y68F releases the doubly cut product more slowly than wt SfiI. [For both enzymes, the amount of substrate consumed in the initial burst phase was about 70% of the enzyme concentration rather than 100%: this was as expected,^32^ as the addition of SfiI to a two-site substrate leads not only to DNA with one SfiI tetramer bridging the two sites in *cis* but also to DNA carrying a tetramer at each site, which resists cleavage.^33,35^]

### Cleavage of one-site plasmid

**Fig. 5:**
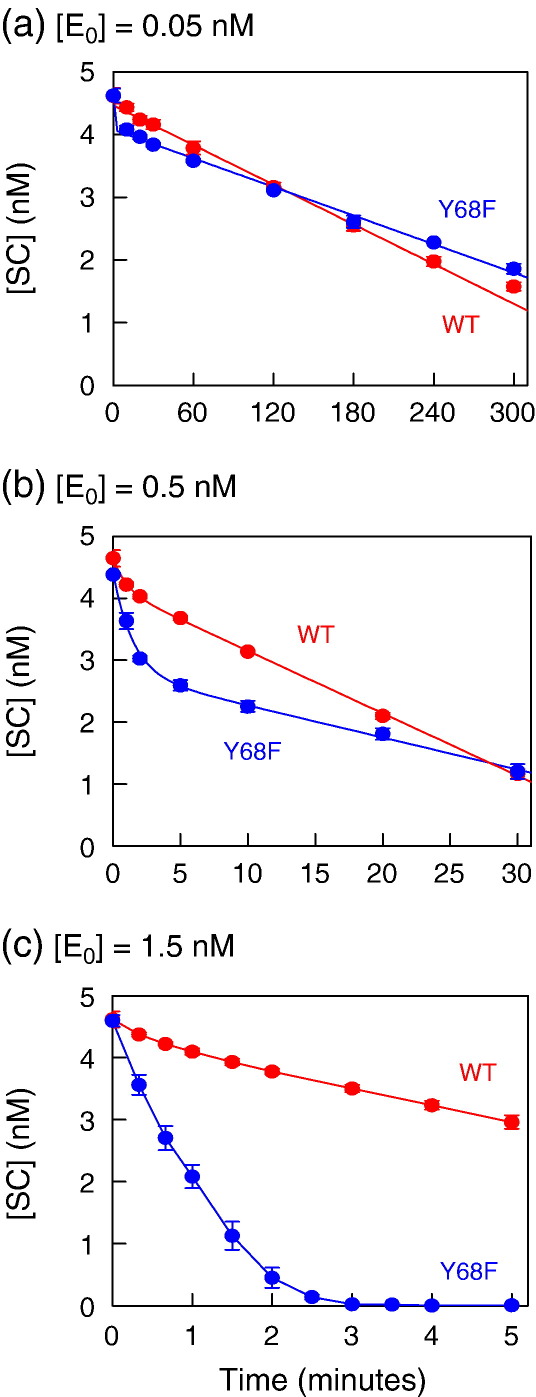
One-site reactions. The reactions contained, in reaction buffer at 50 °C, 5 nM [^3^H]pGB1/S1 (a plasmid with one SfiI site, initially 90–95% SC monomer), and either wt SfiI (red circles) or Y68F (blue circles) at one of the following concentrations: (a) 0.05 nM enzyme; (b) 0.5 nM enzyme; (c) 1.5 nM enzyme. The reactions were carried out and analysed as in Figure 4 to determine the residual concentrations of the SC DNA substrate.

The turnover rate of wt SfiI on a plasmid with one site is typically about 10 times slower than on a plasmid with two sites.^26^^,^^32^^,^^33^ To see if the Y68F mutant behaved like wt, both enzymes were tested against pGB1/S1, a plasmid that carries one of the two SfiI sites from pGB1.^26^ During these reactions, the SC substrate was cleaved directly to the final product, LIN DNA, with a double-strand break at the SfiI site, without liberating the nicked OC form (data not shown): only the decline in the substrate concentration is shown here (Figure 5).

The first tests employed the same enzyme concentration (0.5 nM; Figure 5(b)) as that used for the steady-state reactions on the two-site plasmid (Figure 4(a) and (b)). For wt SfiI, most of the reaction proceeded with a linear decline in the concentration of the substrate with time and, as expected, its reaction velocity was 10-fold lower than that on the two-site plasmid (0.21 compared to 2.2 mol/mol/min). However, the same concentration of Y68F yielded markedly biphasic kinetics: a rapid phase that could be fitted to a single exponential to give a rate constant of 0.7 min^− 1^, followed by a slower linear phase with a velocity of 0.10 mol/mol/min (Figure 5(b)). The amount of substrate consumed during the fast phase (about 1.5 nM) was larger than the amount of enzyme in this reaction (0.5 nM), so the fast phase cannot be due to a pre-steady-state burst of product formation stoichiometric with the enzyme. Instead, the fast phase must reflect multiple turnovers of an active form of the enzyme that decays exponentially to a less active form, the latter being responsible for the slow linear phase of the reaction. The initial rate for the utilisation of the one-site plasmid by Y68F, in the fast phase, was about sevenfold faster than its reaction on the two-site plasmid. Moreover, even during the slow linear phase, Y68F gave virtually the same rates on the one-site and two-site plasmids (0.11 and 0.10 mol/mol/min, respectively).

To see whether the biphasicity varied with enzyme concentrations, both Y68F and wt SfiI were tested against the one-site plasmid at lower (0.05 nM; Figure 5(a)) and at higher (1.5 nM; Figure 5(c)) concentrations. At each level of wt SfiI, the majority of the SC substrate was utilised at a single zero-order rate: the rates increased in direct proportion to the enzyme concentration. Low concentrations of Y68F also gave, for most of the reaction, a linear decline in the concentration of the one-site substrate with time, at a velocity (0.15 mol/mol/min; Figure 5(a)) that was similar to the slow phase of the reaction at 0.5 nM Y68F (0.10 mol/mol/min; Figure 5(b)). In contrast, at high levels of Y68F (Figure 5(c)), virtually all of the one-site substrate was consumed rapidly, at a similar rate to the fast phase from the reaction with 0.5 nM protein.

The active form of Y68F that gives rise to the rapid phase of cutting the DNA with one site is possibly the tetrameric enzyme bound to a single copy of its recognition sequence. This then may decay to a less active form upon binding a second copy to give the synaptic complex, the tetramer bound to two recognition sites. The synaptic complex will form more readily on a two-site DNA than on a one-site DNA,^39^ so both wt and mutant enzymes are likely to cleave DNA with two sites by forming a synaptic complex bridging two sites in *cis*. However, despite the difficulty of synapsing sites in *trans*, wt SfiI cleaves DNA with one site mainly by forming synaptic complexes between sites in separate DNA molecules and not by acting at individual sites.^36^^,^^38^ The complex of wt SfiI bound to one site is therefore either inactive or never present at a significant level. The Y68F mutant may differ from wt in one or both of these aspects.

### Reactions *in trans*

If a restriction enzyme cleaves a plasmid with a single target by acting at that target alone, then the addition of an oligoduplex carrying the recognition sequence will reduce the rate at which that enzyme cleaves the plasmid, as the duplex will act as a competitive inhibitor.^20^^,^^44^ On the other hand, a restriction enzyme that cleaves plasmids with a single site by spanning two sites in *trans* will be activated by adding an appropriate concentration of the specific duplex, as the synaptic complex can then be formed more readily with one molecule of plasmid and one molecule of duplex rather than with two molecules of plasmid.^17,26,28,30^ [Higher concentrations of duplex will, however, lead to synaptic complexes with two molecules of the duplex and thus inhibit plasmid cleavage.] Hence, if the fast phase of the reaction of Y68F is due to its reaction at an individual site, it should be inhibited by adding even low concentrations of cognate duplex. But if it is due to a reaction in *trans* spanning separate molecules of DNA, it should be activated by low levels of the duplex.

**Fig. 6:**
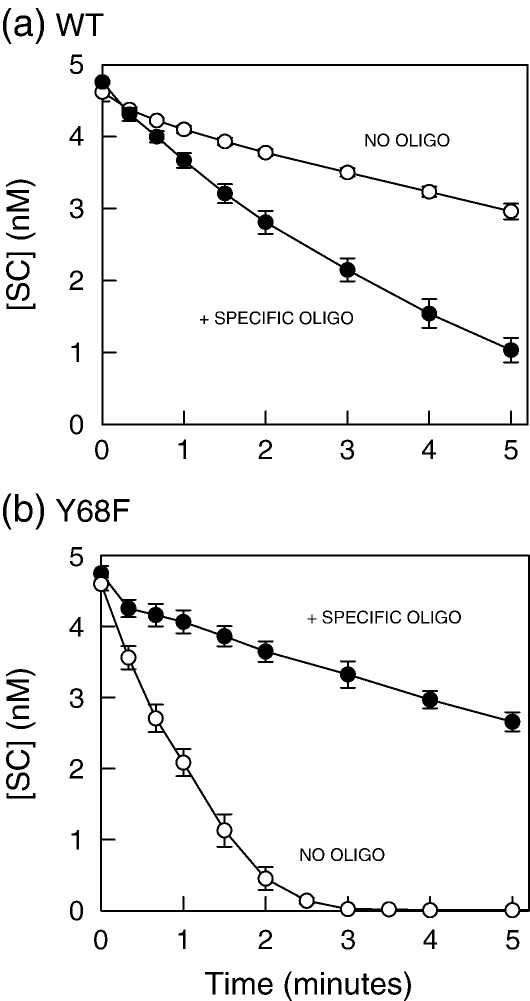
Reactions *in trans.* The reactions contained, in reaction buffer at 50 °C, 5 nM [^3^H]pGB1/S1 (initially 90–95% SC monomer), 1.5 nM enzyme ((a) wt SfiI; (b)Y68F) and either 25 nM HEX-21 (filled circles) or no oligoduplex (open circles). [HEX-21 is a specific duplex for SfiI; Table 1.] Reactions were carried out and analysed as in Figure 4 to determine the residual concentrations of the SC DNA substrate.

Previous experiments had shown that 25 nM duplex activated wt SfiI against 5 nM one-site plasmid.^26^ This amount of duplex was therefore added to the reaction of Y68F on a one-site plasmid and, for comparison, the wt reaction (Figure 6). HEX-21 (Table 1) was again used as the duplex. Relatively high enzyme concentrations were employed so that Y68F cleaved almost all of the DNA in the fast phase (Figure 5(c)). The plasmid with one site was cleaved by wt SfiI more rapidly in the presence of HEX-21 than in its absence (Figure 6(a)). In marked contrast, the fast phase of the reaction of Y68F on the one-site plasmid was much slower in the presence of HEX-21 than in its absence (Figure 6(b)). In further experiments at this high concentration of Y68F, activated cleavage of the one-site plasmid was not observed at any level of duplex tested (from 1 nM to 1 μM). However, at 0.05 nM Y68F, an enzyme concentration that results in most of the one-site plasmid being cleaved in the slow phase (Figure 5(a)), the addition of HEX-21 enhanced the reaction rate (data not shown).

It thus seems likely that the fast phase of the reaction of Y68F on the plasmid with one SfiI site is indeed due to the tetrameric protein bound to an individual site, while the slow phase of the Y68F reaction and the monophasic reaction of wt SfiI on the one-site DNA are both due to a tetramer bound to two sites in *trans*. If so, the Y68F mutant bound to one SfiI site gives a much higher reaction velocity than wt SfiI at a solitary site. However, if a reaction in free solution is carried out by adding a one-site DNA to a protein that can bind two sites, then, as there exists no physical means to prevent the protein from binding two separate molecules of DNA, one can never completely exclude the possibility that the observed reaction involves the protein bridging sites in *trans*.^41^^,^^42^

### Reactions at individual sites

To determine unequivocally whether a protein capable of binding two DNA sites is active after binding just one, the protein must be barred from contacting two DNA molecules at the same time. This can be achieved by immobilising the DNA on a solid surface at a low density, so as to hold the DNA molecules separate from each other.^41^^,^^42^ This strategy was applied to SfiI by using a 30-bp duplex with a single cognate site, BIO-30 (Table 1). The site in this duplex is the same as that in the one-site plasmid pGB1/S1 with respect to spacer and flanking sequences. The duplex carried a biotin tag on the 5′ end of one strand, and this was used to attach it to a streptavidin-coated bead. The ratio of streptavidin on the bead to biotinylated DNA was set at 125:1, conditions that result in the mean distance between adjacent DNA molecules on the surface of the bead being considerably longer than that between the two DNA-binding clefts in the SfiI tetramer. This leaves the individual DNA chains too far apart to allow SfiI to form a synaptic complex with two duplexes. The BIO-30 duplex also contained a ^32^P label at the 5′ end of the other strand, so that cleavage of this strand could be monitored. These experiments employed enzyme concentrations in excess of immobilised DNA and thus are, effectively, single-turnover reactions. The observed rates of DNA cleavage thus reflect the phosphodiester hydrolysis step in the reaction pathway.

**Fig. 7:**
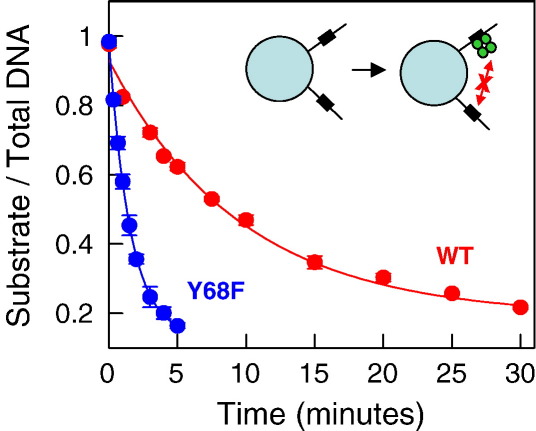
Immobilised oligonucleotides. Samples at 50 °C contained, in reaction buffer without MgCl_2_, SfiI protein (50 nM) and ^32^P-labelled BIO-30 (final concentration, 1 nM) that had been previously bound to streptavidin-coated magnetic beads (125 nM in streptavidin). The cartoon shows two specific duplexes (rectangles to mark recognition sites) bound to a bead (cyan circle) that are too far apart to allow the SfiI tetramer (green circles) to bridge the duplexes. The protein was either wt SfiI (red circles) or the Y68F mutant (blue circles). MgCl_2_ was then added to a final concentration of 10 mM. Aliquots were taken at the times indicated on the *x*-axis and analysed as in Materials and Methods to determine the extent of cleavage of the labelled strand of BIO-30. The amount of intact DNA, relative to the total, is given on the *y*-axis. The line through each set of data points is the best fit to a first-order rate constant: for wt SfiI, 0.1 min^−^ ^1^; for Y68F, 0.7 min^−^ ^1^.

Even though wt SfiI generally acts on DNA substrates with a single site by bridging two DNA molecules in *trans* (Figures 3(a) and 6(a)), it was still able to cleave a one-site DNA even when the individual molecules of that DNA were isolated from each other by immobilisation on the bead (Figure 7). The extent of cleavage of the immobilised substrate by wt SfiI was fitted to a single exponential to give an apparent rate constant of 0.1 min^− 1^. This rate constant for phosphodiester hydrolysis by wt SfiI at a single site is very much slower than measured previously for the same process in its synaptic complex with two sites (0.1 s^− 1^).^32^ Even though the immobilised duplex used here differs from the plasmids used previously for reactions in bulk solution, this 60-fold difference is consistent with other studies that have shown that the activity of wt SfiI bound to two sites is ≥ 30 times higher than that at a single site.^36^

The single-turnover reaction of Y68F on the immobilised DNA with one SfiI site also gave an exponential decline in substrate concentration, with an apparent rate constant of 0.7 min^− 1^ (Figure 7), much faster than wt SfiI. Hence, the Y68F mutant bound to a single site cleaves that site more rapidly than wt SfiI at an individual site (Figure 7). Moreover, the rate constant for phosphodiester hydrolysis by Y68F bound to the immobilised DNA equals that from the fast phase of the reaction of Y68F on the one-site plasmid in bulk solution (Figure 5(b)). Although these experiments employed different types of DNA substrates—in one case an oligoduplex attached to a coated bead, and in the other case a plasmid in free solution—they still support the view that the fast phase from the reaction of Y68F on the one-site plasmid is due to the enzyme bound to a single site.

## Discussion

The tetrameric SfiI restriction endonuclease^26^^,^^34^ is now one of the principal experimental systems used for the analysis of long-range interactions between distant DNA sites.^39^ Two of its subunits (a primary dimer)^24^^,^^28^ form one DNA-binding cleft, and the other two form a second cleft on the opposite side of the protein: the clefts are separated by about 80 Å.^25^ SfiI recognises a palindromic DNA sequence^31^ and the two subunits in each primary dimer interact symmetrically with the cognate site, in much the same way as a standard dimeric restriction enzyme with a single DNA-binding cleft. However, virtually no DNA is cleaved by the complex of wt SfiI with one recognition site: instead, almost all of its reactions are due to the complex with two sites.^33^ Moreover, it has to be the scissile site in both clefts: a complex carrying the recognition sequence in one cleft and, in the other cleft, either a noncognate sequence 1 bp different or a phosphorothioate derivative of the correct sequence, fails to cleave either DNA.^36^^,^^38^ Hence, information about the occupancy of each DNA-binding cleft, and the susceptibility of the sequence in each cleft, must somehow be transmitted through the protein to the other cleft 80 Å away.

The transfer of information between the two DNA-binding clefts in a tetrameric restriction enzyme must occur through the interface between the two primary dimers.^41,42^ Inspection of the crystal structure of SfiI bound to two cognate duplexes suggests that Tyr68 may play a pivotal role at this interface (Figure 1). The aromatic ring of this tyrosine sits in a hydrophobic pocket in the opposite subunit, and its hydroxyl group forms an inter-subunit hydrogen bond across the dimer interface.^25^ The removal of the hydroxyl function from Tyr68, by the conservative mutation to phenylalanine, resulted in a protein, Y68F, that retains the tetrameric structure of wt SfiI and its ability to bind two DNA sites at the same time, to cleave two sites in *cis* concertedly (Figures 2, 3 and 4). In these respects, Y68F is like wt SfiI, though its turnover rate on the two-site plasmid is about 20-fold lower than wt, on account of its slow dissociation from the doubly-cut product (Figure 4(c)). However, Y68F differs markedly from wt on DNA with one SfiI site (Figure 5). The wt enzyme acts more slowly on one-site than on two-site DNA because it acts on the one-site DNA in *trans*, bridging two separate molecules of the DNA, and on the two-site DNA in *cis*, looping out the intervening DNA. In contrast, the steady-state reaction of Y68F on the plasmid with one SfiI site gave biphasic kinetics—a fast phase that was considerably faster than its reaction on the two-site plasmid, but which then declined exponentially to a slower phase whose rate matched that on the two-site DNA. Both phases involve multiple turnovers of the enzyme. Further experiments showed that the fast phase is due to Y68F acting at individual sites and that the slower phase is due to its synaptic complex with two DNA sites (Figures 6 and 7). Y68F can thus cleave one-site DNA without the need to bridge two separate molecules of the DNA (Figure 6). The net effect of this Tyr→Phe mutation is thus to switch SfiI from an enzyme showing high activity at two sites and low activity at one site to the exact opposite—low activity at two sites and high activity at one site. It inverts the ratio of its turnover rates on two-site *versus* one-site substrates from 10:1 for wt SfiI to approximately 1:10 for Y68F (as measured from its fast phase on the one-site DNA).

### Kinetic model for SfiI cooperativity

**Fig. 8:**
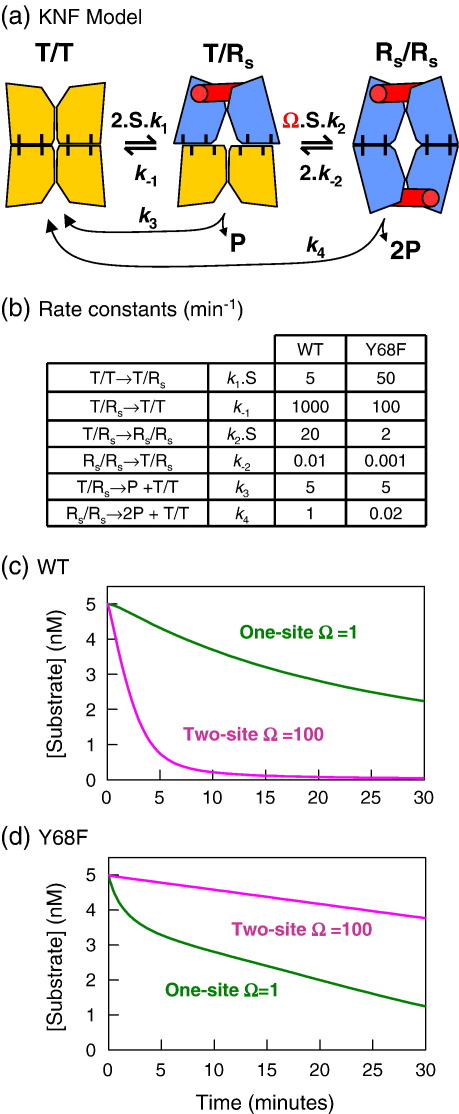
Mechanism for SfiI cooperativity. (a) The scheme indicates three conformational states: the free enzyme, T/T (T indicates the two subunits that form one DNA-binding unit, so the tetrameric protein is noted as T/T); the enzyme after a substrate-induced conformational change at one DNA-binding cleft, T/R_S_; the enzyme after induced changes in both clefts, R_S_/R_S_. The cartoons show the bound DNA as red cylinders and the subunits in the T and R states in yellow and blue, respectively. The T→R_S_ transition is proposed to narrow the DNA-binding cleft and to increase the separation of the subunits at the dimer interface. The interactions between Tyr68 and Gln30 at this interface are indicated by hatch marks. Each step in this scheme is assigned a rate constant, as shown: statistical factors of 2 are applied to *k*_1_ and *k*_− 2_, to account for the two alternative routes for these steps, and a factor (Ω) of 100 is applied to *k*_2_[*S*] for reactions *in cis*. (b–d) The decrease in the concentration of intact DNA with time was calculated for the scheme in (a) by numerical integration using the value indicated in (b) for each rate constant. For all calculations, initial concentrations were [*E*_0_] = 0.5 nM and [*S*_0_] = 5 nM. These values yielded theoretical curves for the reactions of (c) wt SfiI on one-site DNA (green line) and two-site DNA (purple line), and (d) Y68F on one-site DNA (green line) and two-site DNA (purple line).

Cooperative action by an oligomeric protein is generally accounted for by either the Monod–Wyman–Changeux (MWC) or the Koshland–Nemethy–Filmer (KNF) scheme.^51^^,^^52^ In both schemes, each subunit of the oligomer exists in either an inactive low-affinity tense (T) state or an active high-affinity relaxed (R) state. In the following, the terms T and R apply to a primary dimer of SfiI, a single DNA-binding unit, so that the tetramer is noted as T/T, T/R or R/R (Figure 8(a)): the subscript “S” will denote bound substrate. In the MWC model, the T→R transition occurs in all or none of the subunits so that the protein never contains both T and R subunits at the same time. On the other hand, the KNF model proposes sequential T→R transitions upon ligand binding to each subunit, allowing hybrid oligomers containing both T and R_S_ subunits. [To keep the Y68F protein active, the reactions reported here were carried out by adding Mg^2+^ to mixtures of enzyme and DNA and thus might appear to start from an enzyme–DNA complex rather than from free enzyme. However, these reactions had the same kinetics as those started by adding enzyme to the DNA–Mg^2+^ mix, so the dissociation of any DNA bound in the pre-equilibrium must be rapid compared to the subsequent steps. Hence, these reactions can be considered as starting from the free enzyme. In the absence of metal ions as in the pre-equilibria, SfiI binds weakly and nonspecifically to DNA.^36^]

The properties of wt SfiI, its cooperative binding to two DNA duplexes and its enhanced activity on two-site over one-site substrates, can be accounted for readily on either the MWC or the KNF model. The wt enzyme almost always needs to bind two copies of its recognition site before it can cleave DNA; thus, on DNA with one site, it acts primarily *in trans*, bridging two DNA molecules. The wt reaction on a one-site DNA thus proceeds largely via the R_S_/R_S_ state, to the virtual exclusion of T/R_S_ or R/R_S_ forms. On a DNA with two sites, the wt enzyme binds both sites *in cis* to again yield the R_S_/R_S_ state, but in this case the transition from the free T/T form to the doubly-liganded R_S_/R_S_ structure will occur much more readily than on a one-site DNA due to the local concentration effect for sites *in cis*.^39^^,^^45^ In contrast, the Y68F mutant of SfiI cleaves DNA readily after binding just one site, so that a substrate-induced conformational change in one primary dimer, to give the hybrid T/R_S_ state, is sufficient for Y68F activity. The presence of hybrid species is allowed by the KNF scheme but not by the MWC scheme; thus, if both Y68F and wt SfiI follow the same mechanism, it has to be a KNF scheme.

A simplified three-state version of the KNF model for cooperative action by a dimer, as opposed to the full nine-state version, is shown in Figure 8(a): in this scheme, the substrate-binding and the T→R steps always occur together so that the unliganded T/R and R/R states, the partially liganded T/T_S_ and R/R_S_ states and the fully liganded T_S_/T_S_ and T_S_/R_S_ forms are all excluded, leaving only T/T, T/R_S_ and R_S_/R_S_. It also coalesces the DNA-cleavage and product-release steps.

To examine whether the scheme in Figure 8(a) can describe the reactions of both wt and Y68F enzymes on both one-site and two-site substrates, the differential equations for the change in concentration of each species with time were solved by numerical integration, using a range of different values for each of the six rate constants: *k*_1_ and *k*_− 1_ for the first DNA-binding event; *k*_2_ and *k*_− 2_ for the second DNA-binding event; and *k*_3_ and *k*_4_ for DNA cleavage/product release from the singly- and doubly-liganded protein. Numerous trials were undertaken, with different values, until a match between model and experiment had been obtained. The values used for Y68F and for wt SfiI differed from each other, but in both cases, the same sets of rate constants were used for their reactions on the one-site and two-site substrates. The distinction between one and two sites was modelled instead by multiplying *k*_2_[S], the association rate for the second DNA, by a factor Ω to account for the local concentration of one DNA site in the vicinity of another being higher for sites in *cis* than for sites in *trans*: by definition, Ω = 1 for sites in *trans* and, by selection, Ω = 100 for sites in *cis*. Even though the latter value was selected arbitrarily, it reflects experimental measurements^45^ and large (10-fold) variations in this figure made essentially no difference to these calculations (data not shown).

For both enzymes, a set of rate constants was found that yielded progress curves that closely matched the experimental data for that enzyme on both one-site and two-site substrates (Figure 8(c)) for wt; Figure 8(d) for Y68F: the corresponding experimental data are shown in Figures 4 and 5. To match the experimental data, it was necessary to stipulate that both rate constants leading to the formation of T/R_S_ (*k*_1_ and *k*_− 2_) were smaller for wt SfiI than for Y68F and that, conversely, both rate constants leading away from T/R_S_ (*k*_− 1_ and *k*_2_) had to be smaller for Y68F. In particular, for Y68F, *k*_2_ had to be held at a low value, comparable to that for the DNA-cleavage/product-release steps from the T/R_S_ intermediate (*k*_3_), in order to simulate the biphasic reaction profile on the one-site DNA (Figure 5(b)). [If *k*_2_ was set either much smaller or much larger than *k*_3_, the substrate concentration declined linearly with time. In addition, in order to account for the slower turnover rate of Y68F on the two-site plasmid, *k*_4_ had to be fixed at a lower value than for wt SfiI, but this concurs with the experimental data: *k*_4_ encompasses both DNA cleavage by R_S_/R_S_ and product release, and the release of the doubly-cut product is slower from Y68F than from wt SfiI (Figure 4(c)).] However, the models used the same values for *k*_3_, the rate constant for DNA cleavage by the complex with one DNA (the T/R_S_ state). The fact that the T/R_S_ state leads to some DNA cleavage by Y68F, but virtually none by wt SfiI, is due to the differing extents to which this state is populated during their reactions.

In this scheme (Figure 8(a)), the principal effect of replacing Tyr68 with Phe is to reduce the differences in free energy between the T/R_S_ state and either the T/T or the R_S_/R_S_ structures. With wt SfiI, the T/R_S_ state is very strongly disfavoured relative to either T/T or R_S_/R_S_ in thermodynamic terms, and it also has a short lifetime so that wt SfiI cleaves DNA with one site by forming the R_S_/R_S_ complex with two sites *in trans*. Even when a high value is allotted to the wt enzyme for *k*_3_ (the rate constant for DNA cleavage by the T/R_S_ state), virtually all of the DNA is still cleaved by wt via its R_S_/R_S_ state. In contrast, the T/R_S_ state for Y68F is more stable thermodynamically than that for the wt enzyme and it also has a longer lifetime, due to a low value for *k*_2_, the rate constant for the T/R_S_→R_S_/R_S_ step. Hence, with Y68F, the one-site DNA is cleaved initially via the T/R_S_ state, but because the T/R_S_→R_S_/R_S_ transition still has a negative Δ*G*°, it will eventually form the R_S_/R_S_ state with two DNA molecules in *trans*.

### Structural model for SfiI cooperativity

How does this Tyr→Phe change stabilise the T/R_S_ intermediate? At present, a crystal structure is available for wt SfiI bound to two cognate duplexes, but not for the free protein without DNA. Given the scheme in Figure 8(a), the solved structure is presumably akin to the R_S_/R_S_ state, although it may denote a precursor to the catalytically active form.^25^ Nevertheless, the cartoon of R_S_/R_S_ (Figure 8(a)) relates to the known crystal structure (Figure 1(a)). In this structure, the two subunits that make up the primary dimers are in close proximity at the DNA-binding surface but lie distant from each other at the dimer interface.

In almost every case to date where crystal structures are available for a restriction enzyme in both DNA-bound and free forms,^1^^,^^4^^,^^5^ the DNA-binding cleft has an open configuration in the absence of DNA, but it closes up around the DNA in the complex. Hence, it is possible that the two DNA-binding clefts in SfiI also have open configurations in the absence of DNA but become narrower upon binding cognate DNA. If so, then the movement of two subunits towards each other across the DNA-binding cleft might result, given rigid body motion, in these subunits moving away from each other at the interface with the other primary dimer (as illustrated by the T/T→ R_S_/R_S_ transition in Figure 8(a)). The interface between the dimers is relatively flat and is almost entirely hydrophobic in nature,^25^ which might permit each subunit to slide or rotate past the opposite subunit in the other dimer. A similar scheme for intersubunit motion has been proposed for the subunit rotation step in site-specific recombination by γδ resolvase.^53^ However, in the crystal structure of wt SfiI bound to two duplexes, the hydroxyl groups of Tyr68 in all four subunits make direct hydrogen bonds to Gln30 in their partner subunits across the dimer interface. In order to satisfy the hydrogen-bonding potentials of both Tyr68 and Gln30, these interactions are likely to be present not only in the R_S_/R_S_ state, as observed in the crystal structure, but also in the free protein, the T/T state, even though the free protein may have a different geometry at the dimer interface. On the other hand, when only one of the two primary dimers changes conformation from T to R upon DNA binding, the Tyr68 and Gln30 residues in the liganded R_S_ dimer will no longer be in register with those in the unliganded T dimer (Figure 8(a)). This dislocation may destabilise the T/R_S_ state, as neither Tyr68 nor Gln30 will have a suitably positioned hydrogen-bonding partner.

In the above scheme, one function of Tyr68 in wt SfiI is to ensure that whenever one of the two dimers binds DNA and undergoes the T-to-R_S_ transition, the other dimer is forced to follow suit and to undergo immediately its own switch from T to R, so as not to accumulate the highly unstable T/R_S_ state. However, without the Tyr68–Gln30 interaction, as in the Y68F mutant, the T-to-R_S_ transition in one dimer would no longer compel the other dimer to undergo straightaway its own T-to-R switch. The hybrid T/R_S_ form is more stable and has a longer lifetime in the reaction of Y68F compared to wt, so it accumulates sufficiently and exists long enough to allow for some DNA cleavage events while in this state.

## Materials and Methods

### Mutagenesis

Plasmids containing the genes coding for the SfiI restriction endonuclease, pRRS-SfiIR^+^, and the SfiI modification methyltransferase, pSYX33-SfiIM^+^, were provided by Ira Schildkraut (New England Biolabs) and were used to transform *Escherichia coli* ER2353, first with pSYX33-SfiIM^+^ and then with pRRS-SfiIR^+^. The mixture of plasmids isolated from this strain was employed for site-directed mutagenesis of the SfiI restriction gene by the QuikChange method (Stratagene). The resultant PCR products were used to transform *E. coli* ER2353 (pSYX33-SfiIM^+^). The plasmids were isolated from the transformants, and the derivatives of pRRS-SfiIR^+^ were sequenced across the entire gene for the mutant SfiI endonuclease (University of Dundee Sequencing Service): only the Y68F mutation had been introduced.

### Proteins and DNA

Wt SfiI and Y68F were purified from *E. coli* ER2238 cells that had been transformed successively with pSYX33-SfiIM^+^ and pRRS-SfiIR^+^ (or a derivative) as described previously.^26^ The purified enzymes were stored at − 20 °C. Concentrations of wt SfiI and the Y68F mutant were assessed by absorbance at 280 nm using an extinction coefficient of 123,200 M^− 1^ cm^− 1^, where M refers to the tetramer.^34^ All SfiI molarities are thus cited for the tetramer with *M*_r_ = 124,176. Protein structures were analysed with INSIGHT II v. 2005 (Accelrys, San Diego), and surface charge was evaluated with GRASP v. 1.2.^48^

The plasmids pGB1/S1 and pGB1,^26^ which contain one or two SfiI sites, respectively, were used to transform *E. coli* ER2267, and the resulting transformants were grown in M9 minimal media containing 37 MBq/l [*methyl*-^3^H]thymidine (GE Healthcare). The covalently closed form of the plasmid was purified by density gradient centrifugations.^32–35^ The preparations contained mostly the SC form of the monomeric plasmid, with generally < 10% as either dimer or OC. DNA concentrations were assessed by absorbance at 260 nm.

All oligonucleotides were obtained from Sigma Genosys as HPLC-purified samples. Oligonucleotides were annealed to give the duplexes shown in Table 1 by heating a mixture of two oligonucleotides with complementary sequences to 95 °C and then slowly cooling overnight to room temperature. The mixtures generally contained more of the “bottom” strand than the “top” strand to ensure that all of the top strand was incorporated into the duplex. The bottom strand of the BIO-30 duplex (Table 1) was phosphorylated at its 5′ end by using T4 polynucleotide kinase (Roche) and [γ-^32^P]ATP (GE Healthcare), as described before.^30^^,^^36^

### Analytical ultracentrifugation

Sedimentation to equilibrium was done at 20 °C in a Beckman XL-A analytical ultracentrifuge using an An60-Ti rotor with six channel centrepieces.^44^ The three sample channels contained 100 μl of HEX-21 or HEX-21 with either Y68F or wt SfiI in AUC buffer [10 mM Tris–HCl, 50 mM NaCl, 10 mM CaCl_2_ and 1 mM dithiothreitol (DTT); pH 7.9]. The proteins and the DNA, both at 2.5 μM, had been dialysed previously against AUC buffer, and the reference channels contained 110 μl of the dialysate. Centrifugation was carried out sequentially at 10,000, 15,000 and 25,000 rpm. After 16 and 20 h at each speed, the differences in absorbance at 539 nm between samples and references were recorded as a function of centrifugal radius (*r*). At all speeds, the profiles at 16 and 20 h were identical, showing that equilibrium had been reached. Centrifugation was then continued for 8 h at 40,000 rpm to obtain the baseline offset. For each sample, plots of *A*_539_ against centrifugal radius at varied speeds were fitted globally to the equation for a single homogenous species,^49^

$$A_{\text{r}}=A_{0}\text{exp}[M(1-\overset{¯}{\nu }\rho )(r^{2}-r_{0}^{2})(\omega^{2}/2RT)]+B$$

to give values for the molecular mass (*M*, given here as *M*_r_ values): *A*_r_ and *A*_0_ are the absorbances at *r* and at the reference *r*_0_, respectively; ν¯ is the partial specific volume; ρ is the buffer density; ω is the angular velocity; and *B* is the baseline offset. Values for ν¯ and ρ were obtained as before.^44^

### DNA-binding studies

Binding reactions were performed by adding aliquots of wt SfiI or Y68F in dilution buffer to either HEX-21 or HEX-35, or to mixtures of the two duplexes, to give solutions that had, in 20 μl of binding buffer, 5 nM SfiI tetramer and a total duplex concentration of 10 nM. Dilution buffer is composed of 20 mM Tris–HCl, 10 mM β-mercaptoethanol, 0.1 mM EDTA, 10% vol/vol glycerol, 1 mM spermine and 0.2% vol/vol Triton X-100 (pH 7.5). Binding buffer is composed of 20 mM Tris–HCl, 25 mM NaCl, 2 mM CaCl_2_, 5 mM β-mercaptoethanol and 100 μg/ml bovine serum albumin (pH 7.5). After 30 min at room temperature, the samples were mixed with 10 μl of binding buffer containing 4% (wt/vol) Ficoll 400 and applied to 8% polyacrylamide gels in 45 mM Tris–borate (pH 8.3) and 2 mM CaCl_2_, as described previously.^36^ After electrophoresis, the gels were scanned in a Molecular Dynamics PhosphorImager with illumination at 550 nm.

### Enzyme assays

For kinetic experiments, the final reaction mixtures contained 5 nM ^3^H-labelled DNA, either pGB1/S1 or pGB1, and various concentrations of either wt SfiI or the Y68F mutant in 200 μl of reaction buffer at 50 °C. Reaction buffer is composed of 10 mM Tris–HCl, 50 mM NaCl, 10 mM MgCl_2_, 1 mM DTT and 100 mg/ml bovine serum albumin (pH 7.9). The reactions were carried out by adding 10 μl of MgCl_2_ (200 mM) to 190 μl of enzyme and DNA in reaction buffer lacking MgCl_2_ (apart from some trial reactions initiated by adding enzyme to DNA in reaction buffer). Aliquots (15 μl) were removed from the reactions at various times after adding MgCl_2_ (one was removed before adding MgCl_2_ to serve as a zero time point) and vortexed immediately with 10 μl of an EDTA stop mix.^18–20^ The samples were analysed by electrophoresis through agarose under conditions that separate the various products from each other and from the SC substrate. The concentrations of each form at each time point were determined by scintillation counting.^32–36^ All values given here are presented as the means from three independent experiments, with error bars to denote standard errors. Zero-order rates were evaluated using GRAFIT (Erithacus Software) to fit the initial phases of substrate utilisation and/or product formation to linear plots. For some reactions (viz. Figure 5(b)), the concentration of DNA substrate was fitted to an initial exponential decline followed by a zero-order phase. For the various reaction mechanisms considered here (Figure 8), extents of substrate utilisation were modelled by solving the differential equations for the time-dependent changes in the concentrations of each species during the course of the reaction by numerical integration in BERKELEY MADONNA.

Some reactions on pGB1/S1 also contained the oligoduplex HEX-21. These typically contained, in 200 μl of reaction buffer at 50 °C, 1.5 nM enzyme (either Y68F or wt SfiI), 5 nM pGB1/S1 (^3^H-labelled) and 25 nM HEX-21: samples were taken at varied times after initiating the reaction with MgCl_2_ and analysed as described above to determine the amount of pGB1/S1 that had been converted to its linear form.

### Immobilised oligonucleotides

Streptavidin-coated magnetic beads from Promega (10 pmol of streptavidin) were mixed with ^32^P-labelled BIO-30 (0.08 pmol) in 80 μl of SSC,^41^ washed in SSC and resuspended in 200 μl of reaction buffer without MgCl_2_. The requisite concentrations of either wt SfiI or the Y68F mutant were then added before initiating the reactions with MgCl_2_ (final concentration, 10 mM). Aliquots (15 μl) were taken from the reactions at varied times and quenched with loading dye (10 mM NaOH, 100 mM EDTA, 95% formamide, 0.05% bromophenol blue and 0.05% xylene cyanol). After incubating first at 95 °C for 10 min and then on ice for 15 min, the samples were analysed by denaturing gel electrophoresis through a 12% polyacrylamide gel in TBE buffer [45 mM Tris–borate and 1 mM EDTA (pH 8.3)] at ∼ 40 V/cm and 55 °C. The gels were fixed in 20% (vol/vol) acetic acid and 20% (vol/vol) methanol, dried, exposed overnight and scanned in a PhosphorImager. The amounts of intact and cleaved ^32^P strands of the duplex were quantified using ImageQuant (Molecular Dynamics), and the decline in substrate concentration with time fitted to a single exponential in GRAFIT.

## Figures and Tables

**Table 1:**
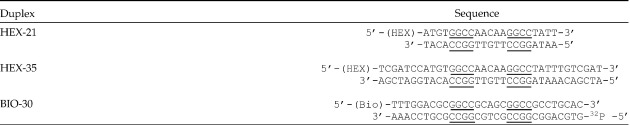
Oligoduplexes

The oligoduplexes HEX-21 and HEX-35 are 21 and 35 bp in length, respectively, and contain the specific recognition sequence for SfiI (underlined). Both are labelled with HEX at their 5′ ends. The oligoduplex BIO-30 is a 30-bp DNA, biotinylated at the 5′ end of the top strand and ^32^P-labelled at the 5′ end of the bottom strand.
